# Depressive Symptom Trajectories and Early Adult Education and Employment: Comparing Longitudinal Cohorts in Canada and the United States

**DOI:** 10.3390/ijerph18084279

**Published:** 2021-04-17

**Authors:** Anita Minh, Ute Bültmann, Sijmen A. Reijneveld, Sander K. R. van Zon, Christopher B. McLeod

**Affiliations:** 1 School of Population and Public Health, University of British Columbia, 2206 E Mall, Vancouver, BC V6T 1Z9, Canada; chris.mcleod@ubc.ca; 2 Community and Occupational Medicine, Department of Health Sciences, University Medical Center Groningen, University of Groningen, Hanzeplein 1, 9713 GZ Groningen, The Netherlands; u.bultmann@umcg.nl (U.B.); s.a.reijneveld@umcg.nl (S.A.R.); s.k.r.van.zon@umcg.nl (S.K.R.v.Z.); 3 Institute for Work & Health, 400 University Ave Suite 1800, Toronto, ON M5G 1S5, Canada

**Keywords:** education, employment, depression, Canada, USA, trajectories

## Abstract

Adolescent depressive symptoms are risk factors for lower education and unemployment in early adulthood. This study examines how the course of symptoms from ages 16–25 influences early adult education and employment in Canada and the USA. Using data from the National Longitudinal Survey of Children and Youth (*n* = 2348) and the National Longitudinal Survey of Youth 79 Child/Young Adult (*n* = 3961), four trajectories (low-stable; increasing; decreasing; and increasing then decreasing, i.e., mid-peak) were linked to five outcomes (working with a post-secondary degree; a high school degree; no degree; in school; and NEET, i.e., not in employment, education, or training). In both countries, increasing, decreasing, and mid-peak trajectories were associated with higher odds of working with low educational credentials, and/or NEET relative to low-stable trajectories. In Canada, however, all trajectories had a higher predicted probability of either being in school or working with a post-secondary degree than the other outcomes; in the USA, all trajectory groups were most likely to be working with a high school degree. Higher depressive symptom levels at various points between adolescent and adulthood are associated with working with low education and NEET in Canada and the USA, but Canadians are more likely to have better education and employment outcomes.

## 1. Introduction

In the transition from school to work, young people face numerous challenges, including obtaining sufficient educational credentials and finding secure full-time employment [1,2,3]. Of particular concern are those neither in employment, education, nor training (NEET), and those entering the labour market with lower educational credentials [4,5]. Both NEET status and low education are associated with poorer long term employment prospects and lifetime earnings [6,7]; higher levels of psychological distress; and higher rates of mental disorders, suicide, and substance use disorders [8,9]. In 2018, 32.8% of young people between the ages 25–34 in the Canada and 50.7% in the USA did not have any post-secondary education [10]. NEET represented 14.8% and 13.4% of young people between the ages 15–29 in 2018, respectively [10].

Young people with histories of poor mental health may be more likely to become NEET or enter the labour market with low education [11]. An estimated 1 in 5 people with major depressive disorder have their first onset before age 25 [12]. Symptoms include pervasive sadness; loss of interest in activities; associated feelings of low self-esteem, suicidal thoughts, or sleep and appetite disturbance; and, among young people, marked irritability [13]. A recent systematic review found that adolescent depressive symptoms increase the risk of failure to complete secondary school; welfare receipt; and educational and labour market disengagement [11]. Cross-sectional studies of Canadians between ages 15 and 29 have shown that past-year depressive symptoms at a clinical level are related to a higher risk of unemployment and NEET status [14,15]. Longitudinal evidence from Australia showed that the onset and the persistence of depressive and anxiety problems are related to a higher likelihood of NEET status in early adulthood [16]. Depressive symptoms have been found to exacerbate school burnout, creating a cycle of emotional disengagement that may have long-term academic and social outcomes [17,18]. Early identification and resources to support the transition from school-to-work for young people with mental health problems may therefore be important intervention strategies.

It remains unclear, however, how the course of depressive symptoms, including changes in frequency and severity from adolescence until early adulthood, influences the risk of NEET or entry into the labour market with lower education. An American study found that young people with mental health trajectories characterized by high levels of internalizing and externalizing problems in either childhood or adolescence had lower educational attainment [19]. A Dutch study found that trajectories characterized by high stable mental health problems in adolescence were associated with NEET or working with low educational credentials at age 20, compared to trajectories of decreasing, or consistently low or moderate, symptoms [20]. However, young people in these studies were only assessed until the ages 20–22, before many transitioned into the labour market. Only one study has examined the relationship between depressive symptom trajectories and young adult education and work outcomes. A recent British study, using data from age 11–24, found that those with early adult onset of symptoms and those with persistent symptoms throughout were less likely to have a university education and, along with those with childhood-limited symptoms, were more likely to be NEET [21]. This evidence suggests that elevated symptoms at any point in the transition from adolescence to young adulthood, not only early or later symptoms, may lead to worse education and employment outcomes.

Differences in the Canadian and American education and labour market systems may influence the magnitude of the association between depressive symptoms and early adult education and employment. While the two countries share similarities, such as high post-secondary enrolment and high labour force demand for credentials [22,23], differences in the level of public funding of education, the range of tuition fees, the presence of private universities, and the presence of post-secondary vocational training may shape the outcome of young people’s school and work trajectories [24,25,26]. Notably, American post-secondary institutions are more stratified with regards to selectivity and resources while Canadian institutions exhibit less inequality, which may translate into smaller mental health inequalities in the Canadian labour market [25]. 

This study compared the relationship between depressive symptom trajectories from age 16 to 25 and early adult education and employment in Canada and the USA. Based on the existing evidence, it was expected that in both countries, trajectories characterized by low-stable symptoms would be associated with a lower likelihood of NEET and working with low educational credentials, relative to trajectories with higher symptom frequency. In Canada, trajectories of higher symptom frequency were expected to have a lower risk of NEET and working with low credentials than their American counterparts. 

## 2. Materials and Methods

### 2.1. Data Sources and Sample Characteristics

We used the longitudinal cohort of the Canadian National Longitudinal Survey of Children and Youth (NLSCY; Cycles 4–8, 2002–2009) [27], and the American National Longitudinal Survey of Youth 1979 Children/Young Adult (NLSY79 Children/YA; 1986–2014), linked to data from the main NLSY79 survey [28]. The target population for the longitudinal cohort of the NLSCY was non-institutionalized children ages 0–11 in 1994 from Canada’s 10 provinces. The target population for the NLSY79 Children/YA was American children of the NLSY79 females. 

Building on earlier work [29], dynamic cohorts were created from each dataset following respondents from age 16–17 until age 24–25 (Canada: *n* = 3357, USA: *n* = 6434). We included individuals with fewer than three time-points of depressive symptom data between ages 16 and 25 to identify depressive symptom trajectory groups (Canada: *n* = 3666, USA: *n* = 6135). To examine the association between depressive symptom trajectories and early adult education and work, we then excluded individuals who did not reach the age of 22 when surveyed (Canada: *n* = 861, USA: *n* = 463) and whose education and employment outcomes were unknown (Canada: *n* = 457, USA: *n* = 1711), resulting in a sample size of 2348 in the Canadian cohort and 3961 in the American cohort.

### 2.2. Measures

#### 2.2.1. Education and Employment Outcomes

Five mutually exclusive categories were defined at respondents’ last follow-up point at age 24 or 25. Those who were currently attending school (including those simultaneously working) were considered (1) in school. Those who were not in school were then classified as (2) working with a high school degree, (3) working with a post-secondary degree, (4) working with no degree, and (5) NEET (neither attending school nor working). 

#### 2.2.2. Depressive Symptoms

Depressive symptoms were measured using a composite of items from the Centre for Epidemiological Studies on Depression scale (CES-D). The CES-D captures the past-week frequency of symptoms across multiple diagnostic categories [30,31]. Five items that appeared in both data sources were used: (1) I did not feel like eating; my appetite was poor; (2) I had trouble keeping my mind on what I was doing; (3) I felt depressed; (4) I felt that everything I did was an effort; (5) my sleep was restless. Respondents rated the frequency of their symptoms on a four-point scale, from “rarely or none of the time (less than 1 day)” to “most or all of the time (5–7 days)”. Scores on the composite scale spanned 0–15, with higher scores indicating higher frequency (Canada mean score at age 16 = 3.94, SD = 2.86, USA mean score at age 16 = 3.51, SD = 2.76). Confirmatory factor analyses for the five items in this study supported a single-factor model at each age in both cohorts (Confirmatory Factor Index: 0.942–0.998, Tucker Lewis Index: 0.884–0.997, Root Mean Square Error of Approximation: 0.011–0.075) [32]. The internal reliability (Cronbach’s alpha) of the scale ranged from 0.55–0.60, which may be considered an acceptable range given the number of items [33].

#### 2.2.3. Confounders

We controlled for variables indicating whether the respondent cohabitated with a spouse/partner, had children, childhood family income (income quartile in the original survey sample), parental education (less than high school/high school/more than high school), parental unemployment in childhood, respondent’s sex (Canada: 49.6% male/50.4% female; USA: 51.5% male, 48.5% female), age, ethnicity (USA: Hispanic/Black/non-Black, non-Hispanic; Canada: Visible Minority/White), mother’s immigration history (American- or Canadian-born/born outside the USA or Canada), single-parent household in childhood, region of residence (USA: Northeast/North Central/South/West; Canada: British Columbia/Alberta/Saskatchewan and Manitoba/Ontario/Quebec/Maritime provinces), urbanicity, and mother’s age. See supplementary file for details on the comparability of the variable derivation across cohorts.

### 2.3. Data Handling and Analyses

First, growth mixture modeling was used to identify depressive symptom trajectories form age 16 to 25 in each of the Canadian and American cohorts [34]. Details about the procedure have been published in Minh et al. [29]. In short, the number of trajectories was decided on by comparing fit statistics (AIC and adjusted BIC), average posterior probability values, and group size, with a priori expectations. Respondents were assigned to a trajectory group, representing subgroups with different average growth curves of depressive symptom frequency between age 16–25, based on respondents’ highest probability of group membership determined by the results of the growth mixture models. 

Next, we described the distribution of the trajectories and other characteristics of the Canadian and American cohorts overall, and by outcome category, testing differences with chi-squared tests. We used multivariable multinomial logistic regressions to estimate the effect of depressive symptom trajectory group membership on education and employment. Using marginal standardization, we calculated the adjusted predicted probability for each outcome category. The resulting predictive probability may be interpreted as the proportion of observations that would have been observed with the outcome if a particular exposure level was imposed on the entire study population [35]. All analyses were weighted using sampling probability weights to account for survey design. Analyses were performed in Stata versions 12 (USA) and 13 (Canada). 

## 3. Results

### 3.1. Distribution of Depressive Symptom Trajectories and Early Adult Education and Employment in Canada and the USA

**Figure 1:**
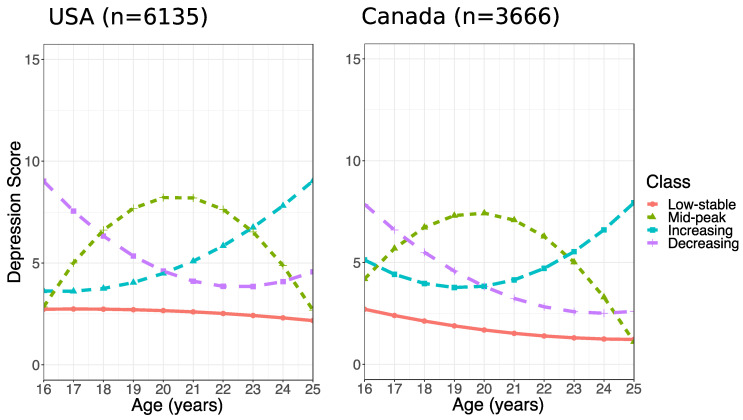
Four estimated trajectories of depressive symptomatology in the United States (left) and Canada (right). CES-D, Center for Epidemiologic Studies Depression Scale. This figure was published in the Journal of Adolescent Health, Vol. 68, Minh, A.; Bültmann, U.; Reijneveld, S. A.; van Zon, S. K. R.; and McLeod, C. B. Childhood Socioeconomic Status and Depressive Symptom Trajectories in the Transition to Adulthood in the United States and Canada. 161–168. Copyright Elsevier (2021).

Four depressive symptom trajectories from age 16–25 were identified in each of the Canadian and American cohorts (see Figure 1): (1) low-stable symptoms, (2) increasing symptoms, (3) decreasing symptoms, and (4) increasing then decreasing symptoms (mid-peak). Table S2 in the Supplementary Materials describes parameter estimates for each trajectory. Further details have been reported in Minh et al. [29].

Table 1 shows the characteristics of the Canadian and American samples. There were some baseline differences across the two cohorts. In the Canadian cohort, 37.5% of young people came from families whose incomes were in the top quartile compared with 29.0% in the American cohort. In the American cohort, 48.0% of respondents’ parents had more than high school education compared with 83.0% in the Canadian cohort. 

In the Canadian cohort, 63.5% of young people had a low-stable depressive-symptom trajectory compared to 77.5% in the American cohort (Table 1). In the Canadian cohort, 11.4% were working with a high school degree and 39.8% were working with a post-secondary degree, compared with 35.6% and 11.4% in the American cohort. NEET represented 4.0% of young people in the Canadian cohort and 5.7% in the American cohort. 

In both cohorts, young people’s education and employment outcomes differed by trajectory (Table 1). For descriptive results, the ‘working with no degree’ and NEET categories were combined in the Canadian cohort to adhere to Statistics Canada reporting guidelines on minimum cell sizes [36]. In both cohorts, there were smaller proportions of the low-stable symptom trajectory and greater proportions of all other trajectories among those working with a high school degree, working with no degree and/or NEET, and in school.

### 3.2. Association between Depressive Symptom Trajectories and Early Adult Education and Employment

Results of the multinomial regression show that in both cohorts, the odds of working with a high school degree relative to working with a post-secondary degree were higher for the increasing (Canada: OR = 3.36, 95%CI: 1.74–6.47; USA: OR = 2.36, 95%CI: 1.49–3.73) and mid-peak trajectories (Canada: OR = 2.74, 95%CI: 1.22–6.12; USA: OR = 2.14, 95%CI: 1.26–3.62) than for the low-stable trajectory, even after adjusting for covariates (Table 2). In both cohorts, the odds of working with a high school degree were also higher for the decreasing trajectory; however, the 95% confidence interval contains the null in the Canadian cohort (USA: OR = 1.59, 95%CI: 1.01–2.49; Canada: OR = 1.60, 95%CI: 0.96–2.70). 

In both cohorts, the odds of working with no degree were higher for the increasing (Canada: OR = 2.94, 95%CI: 1.42–6.06; USA: OR = 4.68, 95%CI: 2.60–8.41) and decreasing trajectories (Canada: OR = 2.10, 95%CI: 1.22–3.61; USA: OR = 2.03, 95%CI: 1.06–3.87). In the American but not the Canadian cohort, the odds of working with no degree were also higher for the mid-peak trajectory (OR = 4.88, 95%CI: 2.52–9.47).

In both cohorts, the odds of NEET were higher for the increasing trajectory (Canada: OR = 3.88, 95%CI: 1.44–10.44; USA: OR = 2.91, 95%CI: 1.56–5.42). 

Finally, the odds of being in school were higher for the increasing trajectory (Canada: OR = 2.30, 95%CI: 1.34–3.93; USA: OR = 2.65, 95%CI: 1.66–4.24). In the American but not the Canadian cohort, however, the mid-peak trajectory was associated with higher odds of being in school (OR = 1.97, 95%CI: 1.10–3.51)

**Figure 2:**
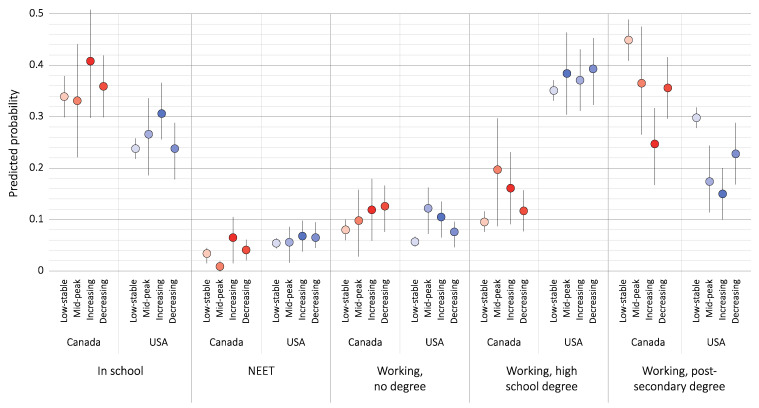
Predicted probability of education/employment by depressive symptom trajectory and country, adjusted for all covariates.

Estimates of the predicted probability of the outcomes revealed between-country differences (Figure 2). We found that in Canada, the low-stable trajectory group was more likely to be working with a post-secondary degree than any other outcome (44.9%, 95%CI: 40.9–48.9%). The mid-peak and decreasing trajectory groups were about as likely to be working with a post-secondary degree (36.5%, 95%CI: 26.1–46.9%; and 35.6%, 95%CI: 29.6–41.6% respectively) as to be in school (33.1%, 95%CI: 22.3–43.9%; and 35.9%, 95%CI: 29.6–42.4%), and the increasing trajectory was most likely to be in school (40.8%, 95%CI: 31.0–50.6%). In the USA, all trajectory groups were more likely to be working with a high school degree than any other outcome. 

## 4. Discussion

This study found that young people’s depressive symptom trajectories from age 16–25 were associated with their early adult education and employment in both Canada and the USA. In line with our hypotheses, trajectories characterized by higher symptom frequency—the increasing, decreasing, and mid-peak trajectories—were related to working with a high school degree or no degree, and/or with NEET status in both countries. However, we found that Canadians were more likely to have better early adult education and employment outcomes than Americans, both amongst those with low-stable symptoms and those with trajectories with higher symptom frequency.

We observed elevated odds of working with high school credentials for the mid-peak, increasing, and decreasing trajectories, in both countries. We also observed higher odds of working with no degree for the increasing and decreasing trajectories in both countries, and higher odds of NEET for the increasing trajectory. These findings are consistent with previous research showing that depressive symptoms between adolescence and young adulthood increase risks of school drop-out and post-secondary enrolment, and unemployment [11,37]. In the transition to adulthood, the rise in levels of negative emotions follows a similar trajectory to the rise in the number stressors facing young people, including developmental challenges (e.g., identity formation) and normative difficulties (e.g., peer conflicts, transitioning out of school), which may result in depressive symptoms [38]. Stress from depressive symptoms may subsequently exacerbate problems such as school burnout [17,39], disrupt social interactions, and compromise academic and work performance [40], making it less likely that young people will pursue further education and more likely they will be unemployed. 

Our study additionally shows that the timing and course of symptoms may be related to early adult education and employment. For example, we observed a relationship between both the increasing and mid-peak trajectories, working with high school or no degree, and NEET. The results likely reflect the negative impact that higher symptom levels between ages 19–25 have on post-secondary completion and employment [11,41]. As well, findings may reflect the negative mental health effects of school dropout, fewer labour market opportunities, and poorer working conditions [42].

The association between the decreasing trajectory and working with a high school or with no degree, on the other hand, suggests that elevated depressive symptoms in adolescence may have long-term impacts, despite decreasing over time. Similarly, using data from Avon county in the United Kingdom, López-López et al. found that young people who display symptoms at some point between ages 17 and 22, regardless of whether or not they improved, had lower odds of attaining a university degree than the low-stable group [21]. Such findings support a sensitive period hypothesis, which suggests that depressive symptoms at a particular developmental stage may have consequences in early adulthood, regardless of the trajectory, because they coincide with key transition periods for education and employment [43,44].

While relative differences in education and employment by depressive symptom trajectories were similar between the two countries, absolute estimates of risk suggest better early adult education and employment outcomes for Canadians with higher symptom trajectories. We found that in Canada, those with increasing symptom trajectories were more likely to be in school, whereas the same group in the USA was most likely to be working with high school credentials. As well, in Canada, those with mid-peak and decreasing trajectories were just as likely to be in school as working with a post-secondary degree, while in the USA, the same groups were most likely to be working with a high school degree. Findings are in line with studies showing a stronger link between poor health and lower education in the USA than in Canada [45]. Having depressive symptoms may be an additional barrier to accessing higher education for young people in the USA, where there are comparatively higher costs of post-secondary education due to higher average tuition, a greater prominence of private universities, fewer public institutions in close proximity to disadvantaged areas, and fewer options for non-university post-secondary education [24,46]. Considering that returns to higher education (including earnings, lower risk of unemployment) are greater in the USA than in Canada [47], American young people with depressive symptoms may be at a greater disadvantage later in life. 

This study used the largest available longitudinal population-level data on young people in Canada and the USA to examine how depressive symptom trajectories relate to early adult education and employment. The study controlled for key confounders and used survey weights to account for the sampling frame of each survey. However, study findings should be considered alongside various limitations. First, we used self-reported information, which is subject to reporting bias. Though we used a validated multi-item measure of depressive symptoms across multiple timepoints, reducing the extent that reporting bias impacts on our estimates, future research may want to triangulate data on depressive symptoms with clinical and parental assessments to improve measurement validity. Second, estimates of the association between depressive symptom trajectory and early adult education and employment are thus likely to be underestimated in both cohorts due to differential non-response and selection bias. Approximately 17% of the Canadian cohort and 30% of the American cohort were excluded due to missing data on the outcome. In both cohorts, missingness was related to indicators of social disadvantage, including younger mothers and lower parental education and, in the USA, also amongst the increasing trajectory group, suggesting that estimates may be conservative. As well, both data sources failed to adequately sample young people who are incarcerated and therefore NEET. Third, the Canadian sample is more socioeconomically advantaged than the American sample. Between-country differences may thus be overestimated. Replication of this study using matching techniques to improve comparability may be warranted.

## 5. Conclusions

This study has a number of implications. First, findings reinforce the importance of developing interventions for prevention in adolescence as well as interventions for NEET and low-educated workers in early adulthood. Future research may want to examine when and how to intervene in adolescence and early adulthood, as the former is a sensitive period and the latter is a period during which education, employment, and mental health may have contemporaneous effects. Second, this study used a life course perspective that may be built on by future research. Rather than examine depressive symptoms at one time point, or lifetime depression, future research may examine when symptoms occurred and incorporate measures of change to symptom frequency or severity. Further future research should address how treatment, including the use of health and social services and medication, affects adolescent depressive symptom frequency and severity and mitigates their impact on early adult education and work. Finally, this study points to a context-dependent relationship between the evolution of depressive symptoms and early adult education and employment. Given institutional differences between Canada and the USA, researchers and policy makers may want to examine how specific changes to mental health, education, and labour market policy in each country improve the life chances of young people with histories of poor mental health as they transition into adulthood.

## Figures and Tables

**Table 1:** Characteristics of the American and Canadian samples.

	Canada Total (*n* = 2348)	Working, Post-Secondary Degree	Working, High School Degree	Working, No Degree ^a^	NEET ^a,b^	In School	*p*	USA Total (*n* = 3961)	Working, Post-Secondary Degree	Working, High School Degree	Working, No Degree	NEET	In School	*p*
		39.8	11.4	9.5	4.0	35.3			27.5	35.6	6.8	5.7	24.4	
Depressive symptom trajectory							*							***
Low-stable	63.5	70.4	55.3	**55.3**		61.3		77.5	87.2	75.2	60.9	70.7	78.4	
Mid-peak	6.2	5.4	9.9	**5.2**		6.3		5.2	3.1	5.6	10.7	5.8	4.5	
Decreasing	10.5	6.3	15.0	**16.0**		11.9		7.9	5.6	9.4	11.7	11.3	6.8	
Increasing	19.7	17.9	19.8	**23.5**		20.4		9.3	4.1	9.8	16.7	12.3	10.2	
Gender							**							**
Male	49.6	48.4	62.7	62.0	32.5	45.4		51.5	45.4	52.7	61.6	44.7	47.8	
Female	50.4	51.6	37.3	38.0	67.5	54.6		48.5	54.6	47.3	38.4	55.3	52.2	
Childhood income quartile							***							***
Lowest	13.6	11.5	22.0	22.9	20.5	9.9		18.3	8.5	20.3	42.1	30.0	14.9	
Second	21.9	21.6	24.3	29.4	33.8	18.0		24.0	14.7	28.6	29.3	28.6	21.6	
Third	27.0	27.7	28.8	19.7	23.8	28.1		28.7	34.3	27.8	19.2	23.2	26.2	
Highest	37.5	39.2	24.9	28.0	21.9	43.9		29.0	42.5	23.3	9.4	18.2	37.3	
Parental education							***							***
Less than high school	6.1	5.6	8.5	10.3	13.8	3.8		8.6	2.7	7.5	26.3	16.2	4.6	
High school graduation	10.9	13.9	14.1	12.2	3.0	7.1		43.4	32.2	51.3	54.9	45.9	35.9	
More than high school	83.0	80.5	77.4	77.5	83.2	89.1		48.0	64.1	41.2	18.8	37.9	59.5	
Parental unemployment in childhood							*							***
No	78.6	77.6	75.0	73.3	61.7	84.4		77.2	81.2	79.1	65.0	71.1	78.0	
Yes	21.4	22.4	25.0	26.7	38.3	15.6		22.8	18.8	20.9	35.0	28.9	22.0	
Mother born in the US/Canada														
No	15.0	13.5	10.8	14.0	19.8	17.8		4.2	4.3	3.7	5.2	2.8	5.3	
Yes	85.0	86.5	89.2	86.0	80.2	82.2		95.8	95.7	96.3	94.8	97.2	94.7	
Single parent status							*							***
No	87.0	89.2	82.9	76.9	86.6	88.7		74.7	84.0	70.6	59.0	65.3	78.5	
Yes	13.0	10.8	17.1	23.1	13.4	11.3		25.3	16.0	29.4	41.0	34.7	21.5	
Residence in rural area							*							
No	83.2	80.7	80.7	81.9	77.8	87.8		76.2	76.5	74.4	78.2	75.4	78.6	
Yes	16.8	19.3	19.3	18.1	22.2	12.2		23.8	23.5	25.6	21.8	24.6	21.4	

* *p <* 0.02, ** *p* < 0.001, and *** *p <* 0.0001. ^a^ Categories combined in bolded text to comply with Statistics Canada privacy regulation. ^b^ NEET = not in employment, education, or training.

**Table 2:** Odds of education/employment outcomes relative to working with a post-secondary degree (with 95% CIs).

	Model 1 ^a^				Model 2			
	Working, High School Degree	Working, No Degree	NEET ^b^	In School	Working, High School Degree	Working, No Degree	NEET	In School
Canada (*n* = 2348)								
Low-stable (ref)	1.00				1.00			
Mid-peak	2.18 (0.97–2.90)	1.33 (0.59–3.01)	0.62 (0.17–2.26)	1.27 (0.66–2.45)	2.74 (1.22–6.12) *	1.61 (0.68–3.84)	0.29 (0.06–1.41)	1.23 (0.68–2.23)
Increasing	3.39 (1.59–3.25) **	2.67 (1.28–5.57) *	5.71 (2.00–16.34) *	2.44 (1.47–4.06) *	3.36 (1.74–6.47) **	2.94 (1.42–6.06) **	3.88 (1.44–10.44) *	2.30 (1.34–3.93) *
Decreasing	1.57 (0.96–2.54)	1.76 (1.03–3.04) *	1.92 (0.98–3.74)	1.45 (1.00–2.13)	1.60 (0.96–2.70)	2.10 (1.22–3.61) *	1.54 (0.78–3.03)	1.37 (0.92–2.02)
USA (*n* = 3961)								
Low-stable (ref)	1.00				1.00			
Mid-peak	1.99 (1.20–3.32) *	4.58 (2.45–8.56) **	2.15 (0.99–4.67) **	1.71 (0.96–3.05)	2.14 (1.26–3.62) *	4.88 (2.52–9.47) **	2.09 (0.95–4.62)	1.97 (1.10–3.51) *
Increasing	2.67 (1.73–4.12) **	5.62 (3.29–9.62) **	3.55 (1.98–6.47) **	2.88 (1.83–4.55) **	2.36 (1.49–3.73) **	4.68 (2.60–8.41) **	2.91 (1.56–5.42) **	2.65 (1.66–4.24) **
Decreasing	1.78 (1.17–2.69) *	2.56 (1.45–4.49) **	2.21 (1.23–3.98) *	1.43 (0.90–2.26)	1.59 (1.01–2.49) *	2.03 (1.06–3.87) *	1.73 (0.91–3.28)	1.32 (0.83–2.10)

* *p* < 0.05 ** *p* < 0.001 ^a^ Model 1 controlled for the age in years at which the outcome was assessed; Model 2 additionally controlled for whether respondents cohabitated with a partner or spouse, and whether respondent had children of their own, respondent’s sex, race, childhood family income, parental education, household unemployment in childhood, mother’s age, mother’s immigration history, single-parent status in childhood, rural residence, and region of residence. ^b^ NEET = not in employment, education, or training.

## Data Availability

Data from the NLSY79 Child/YA used in this study are publicly available from the NLS Investigator from the U.S. Bureau of Labor Statistics: https://www.nlsinfo.org/ (accessed on 16 October 2019). The NLSCY data that support the findings of this study are available from Statistics Canada, but restrictions apply to the availability of these data, which were used under license for the current study. The data are available from the authors upon request and approval from Statistics Canada.

## Supplementary Materials

The following are available online at https://www.mdpi.com/article/10.3390/ijerph18084279/s1, Table S1. Comparison of variable derivation with the NLSY79 Child/YA and the NLSCY datasets. Table S2. Growth parameters, probabilities of group membership, and proportions for a four-class growth mixture model of depressive symptoms with random intercepts in the United States (top) and Canada (bottom).
