# Supplementary material for: Depressive Symptom Trajectories and Early Adult Education and Employment: Comparing Longitudinal Cohorts in Canada and the United States
Source: Int J Environ Res Public Health. 2021 Apr 17;18(8):4279. doi: 10.3390/ijerph18084279 (PMC8073553; doi:10.3390/ijerph18084279)
Supplement: Supplementary file 1 [file ijerph-18-04279-s001.zip › ijerph-1135803-supplementary.pdf]

Depressive symptom trajectories and early adult education and employment: comparing longitudinal cohorts in Canada and the United States

Supplementary File

Table S1. Comparison of variable derivation with the NLSY79 Child/YA and the NLSCY datasets [a]

MEASURE	NLSY79 VARIABLE(S)	NLSCY VARIABLE(S)	STUDY DEFINITION
**GENDER OF CHILD**	Sex of child (Male/ Female)<br><br>Source: NLSY79 Child/YA	Gender of child (Male/ Female/ Not stated/ Refusal/ Don't know)	Male or Female
**RACE**	Child race/ethnicity is based on the report of the mother and corresponds to the 'Racial/Ethnic Cohort from Screener' in the NLSY79 main Youth. This variable designates the respondent as "Hispanic," "black," or "nonblack/non-Hispanic"<br><br>Source: NLSY79 Child/YA	How would you best describe his race or colour ... (White/ Chinese/ South Asian/ Black/ Native/ Arab/ Filipino/ Asian or South East Asian/ Latin American/ Japanese/ Korean/ Other)<br><br>Response options: Yes/ No/ Refusal<br><br>More than one option is possible	US: Hispanic/ Black/ Non-Black, non-Hispanic<br><br>Canada: White/ Person of Colour (Individuals who responded both White and another race or colour were considered in this study to be a Person of Colour)
**MOTHER'S MIGRATION HISTORY**	Country of birth of mother (In the US/ In another country)<br><br>Source: NLSY79 main	(1) Immigration/ Official language status of the person-most-knowledgeable (PMK)<br>(1) Immigration/ Official language status of the spouse/partner<br>(2) Relationship of the PMK to the child<br>(3) Relationship of the spouse/partner to the child	Mother born in the US or Canada, respectively.<br><br>In Canada, mothers were considered to be born in Canada if they were Canadian by birth (i.e. not Canadian by naturalization)
**SINGLE PARENT HOUSEHOLD**	Since the NLSY79 children eligible for interview in the survey are living at least part-time with their mothers, the mother's main Youth household record is used to describe the cross-sectional composition of the child's household.<br>(1) Is partner of mother present in household of mother?<br>(2) Is spouse of mother present in household of mother?<br><br>Source: NLSY79 Child/YA	Child's parent status – Child lives with: Two parents/ One parent only/ Does not live with a parent/ Don't know/ Refusal	Whether child lives with one parent or two (in the US, specifically whether child lives with mother or both mother and her spouse/partner) in the survey year that the child was age 10 or 11.
**MOTHER'S AGE**	Age of mother at date of child interview<br><br>Source: NLSY79 Child/YA	(1) Age of PMK<br>(2) Age of spouse/ partner of the PMK<br>(3) Relationship of the PMK to the child<br>(4) Relationship of the spouse/partner to the child	Age of the mother in the survey year that children were either age 10 or 11
**RELATIVE INCOME (FAMILY)**	What is your best estimate of the total income before taxes and deductions of all household members from all	Total net family income: These variables provide a composite income figure	Percentile within the entire survey sample in which household income falls, in

	sources in the past 12 months, *that is the total income from all household members, before taxes and deductions?<br><br>This variable contains imputed data.<br><br>Source: NLSY79 main	from a number of income values for household members related to the respondent by blood or marriage.<br>This variable does not contain imputed data	the survey year that children were either age 10 or 11.
**HIGHEST PARENTAL EDUCATION**	(1) Highest grade completed by mother as of May 1 of the survey year<br>(2) Highest grade completed by partner in household<br>(3) Highest grade completed by spouse in household<br><br>Source: NLSY79 Child/YA	(1) Highest level of education obtained by the PMK<br>(2) Highest level of education obtained by the spouse/partner	The highest level of education attained by either the mother or PMK and the spouse/partner in the year that children were either age 10 or 11. Three categories: less than high school/ high school graduation/ more than high school.
**PARENTAL UNEMPLOYMENT**	(1) Number of weeks worked in past calendar year<br>(2) Percent of weeks unaccounted for in calculating weeks worked in past calendar year<br><br>Source: NLSY79 main	(1) Worked for pay in the past 12 months -- PMK<br>(2) Worked for pay in the past 12 months – spouse/partner	Households were considered to have experienced unemployment if neither the child's mother or PMK, or the spouse/partner worked for pay in the past 12 months (defined as any work between 1 and 52 weeks in the US).<br><br>Individuals in the US were coded as missing on this variable if more than 10% of weeks in the calendar year were unaccounted for
**REGION OF RESIDENCE**	Region of current residence (Northeast/ North Central/ South/ West)	Province of residence	US: as the reported region in the survey year that children were either age 10 or 11<br><br>Canada: Six-category variable (Atlantic provinces/ Quebec/ Ontario/ Manitoba and Saskatchewan/ Alberta/ British Columbia). Individuals living in the Northern Territories, and outside Canada were recoded as missing to comply with Statistics Canada guidelines for small cell sizes.
**URBANICITY**	Through 1996, this series was based on the respondent's State and county of residence and the "% urban population" data from the County & City Data Book. From 1998-2002 this item was based on whether the respondent was living in an urbanized area or in area with a population greater than 2,500. Beginning in 2004, this item indicates whether the respondent resides within areas of higher population density i.e. an	Size of are of residence in which child lives, according to the 1996 or 2001 Census.<br><br>Response options:<br>• Rural area<br>• Urban, population < 30,000<br>• Urban, population 30,000 to 99,999<br>• Urban, population 100,000 to 499,999	Urban or rural (based on the survey definition)

	urbanized area or a place with a population of 2500 or more.	• Urban, population 500,000 or over

[a] NLSY79 – National Longitudinal Survey on Youth 1979, NLSCY – National Longitudinal Survey on Children and Youth, YA – Young adult, PMK – Person-most-knowledgeable

Table S2. Growth parameters, probabilities of group membership, and proportions for a four-class growth mixture model of depressive symptoms with random intercepts in the United States (top) and Canada (bottom).

**USA**								
**Parameter**	**Mean**	**LCI**	**UCI**	**Variance**	**LCI**	**UCI**	**Average probability of group membership**	**Sample size (%)**
Low-stable							0.88	4716 (77.6)
Intercept	2.76	2.62	2.88	0.79	0.62	0.95		
Slope	-0.00	-0.07	0.06	0.00	0.00	0.00		
Quadratic	-0.01	-0.01	0.00	0.00	0.00	0.00		
Increasing							0.75	571 (9.3)
Intercept	3.72	3.24	4.20	2.88	2.05	3.70		
Slope	-0.05	-0.32	0.23	0.00	0.00	0.00		
Quadratic	0.07	0.04	0.10	0.00	0.00	0.00		
Decreasing							0.77	486 (7.9)
Intercept	8.98	8.40	9.56	3.20	2.40	3.98		
Slope	-1.54	-1.86	-1.22	0.00	0.00	0.00		
Quadratic	0.12	0.08	0.15	0.00	0.00	0.00		
Mid-peak							0.71	316 (5.2)
Intercept	2.99	2.09	3.89	1.06	0.14	1.98		
Slope	2.35	1.73	2.96	0.00	0.00	0.00		
Quadratic	-0.27	-0.33	-0.20	0.00	0.00	0.00		

**Canada**								
**Parameter**	**Mean**	**LCI**	**UCI**	**Variance**	**LCI**	**UCI**	**Average probability of group membership**	**Sample size (%)**
Low-stable							0.86	2380 (64.9)
Intercept	2.71	2.49	2.92	0.00	0.00	0.00		
Slope	-0.33	-0.43	-0.22	0.00	0.00	0.00		
Quadratic	0.02	0.01	0.03	0.00	0.00	0.00		
Increasing							0.70	342 (9.3)
Intercept	5.12	4.29	5.96	3.59	1.92	5.26		
Slope	-0.83	-1.39	-0.27	0.00	0.00	0.00		
Quadratic	0.13	0.07	0.19	0.00	0.00	0.00		
Decreasing							0.74	700 (19.1)
Intercept	7.86	6.82	8.90	2.98	1.13	4.83		
Slope	-1.35	-1.91	-0.79	0.00	0.00	0.00		
Quadratic	0.09	0.03	0.14	0.00	0.00	0.00		
Mid-peak							0.77	244 (6.7)
Intercept	4.19	3.18	5.20	1.28	0.72	1.83		
Slope	1.73	0.65	2.80	0.00	0.00	0.00		
Quadratic	-0.23	-0.36	-0.10	0.00	0.00	0.00		

Note: LCI/UCI = lower and upper bounds for 95% confidence interval. This table was published in the Journal of Adolescent Health, Vol. 68, Minh, A., Bültmann, U., Reijneveld, S. A., van Zon, S. K. R., & McLeod, C. B. Childhood Socioeconomic Status and Depressive Symptom Trajectories in the Transition to Adulthood in the United States and Canada. 161-168. Copyright Elsevier (2021).
